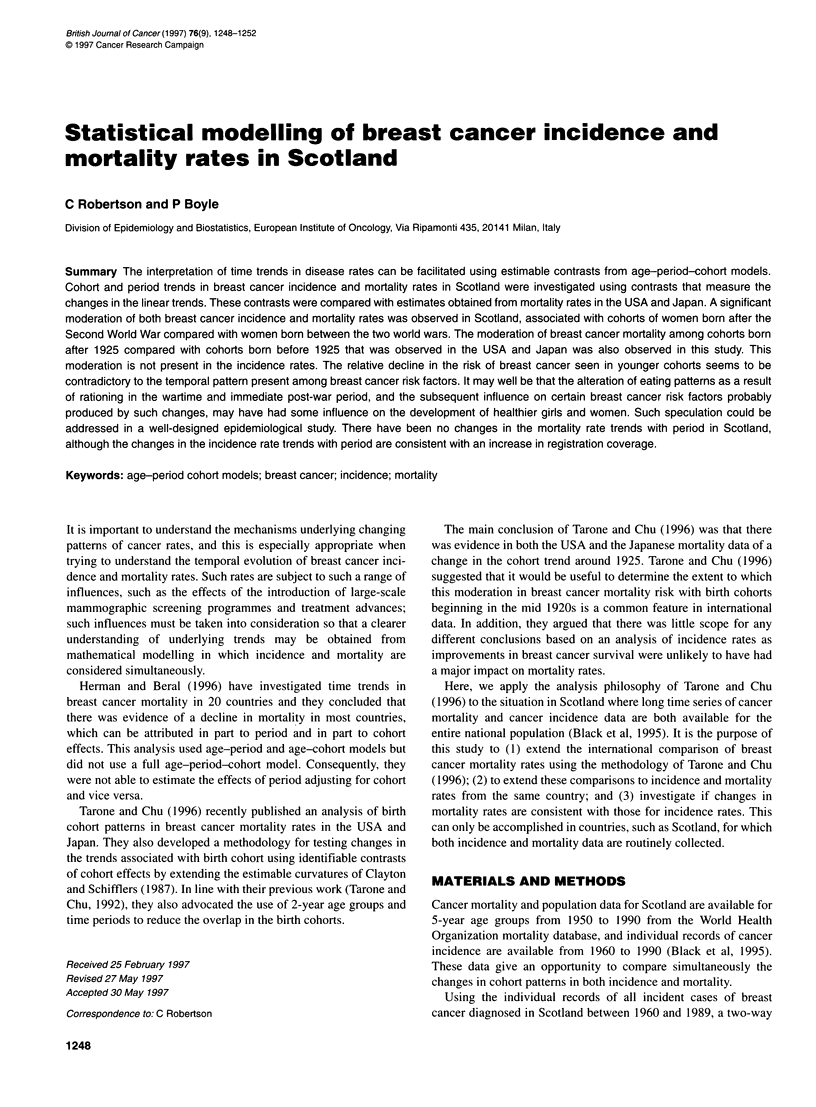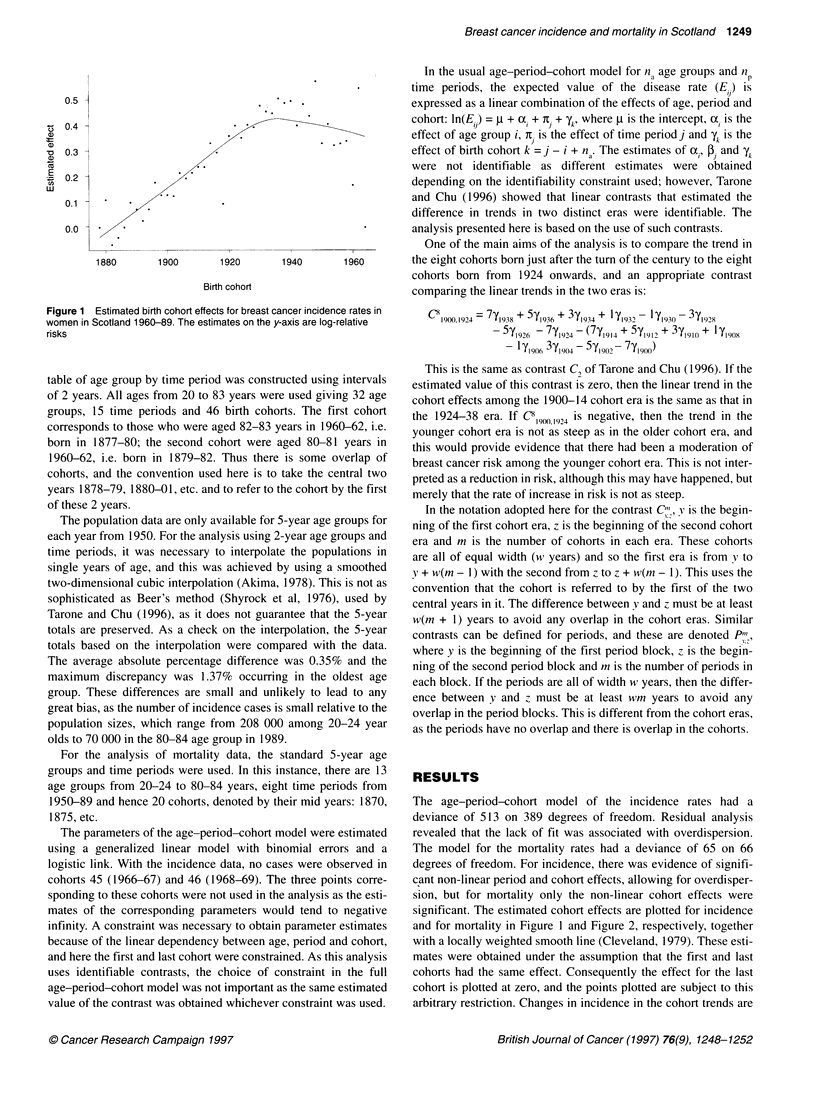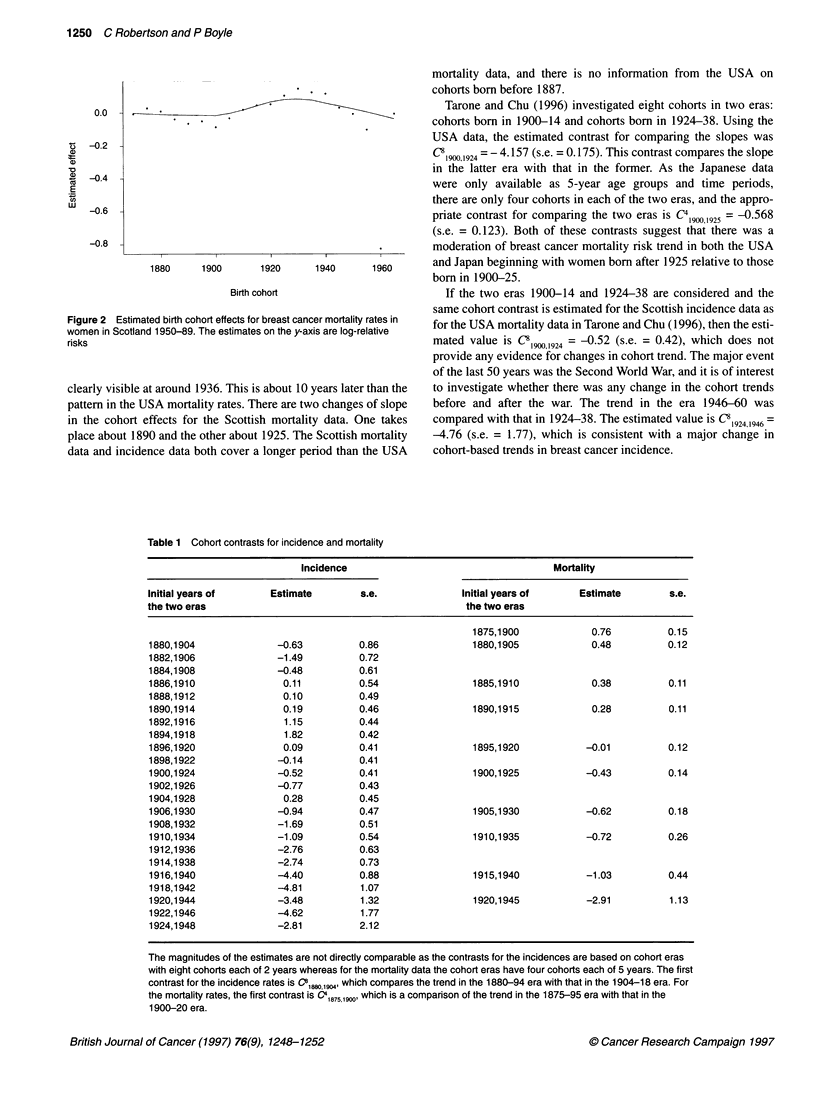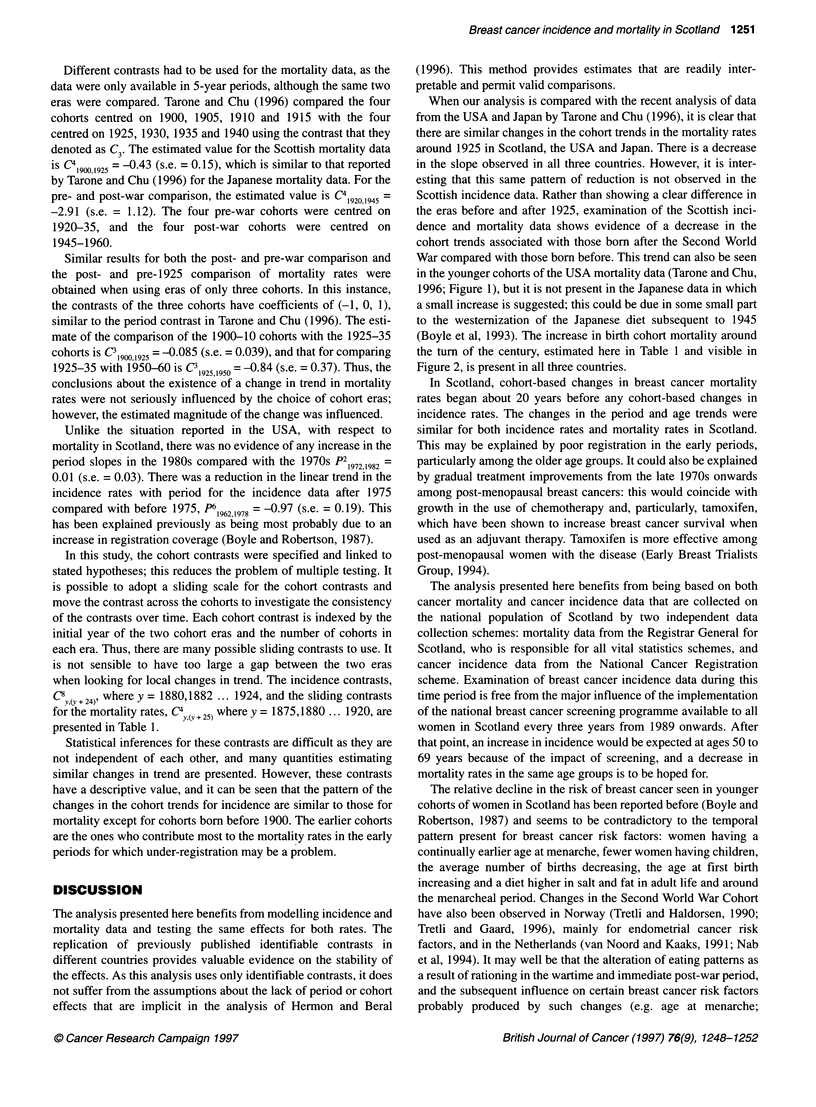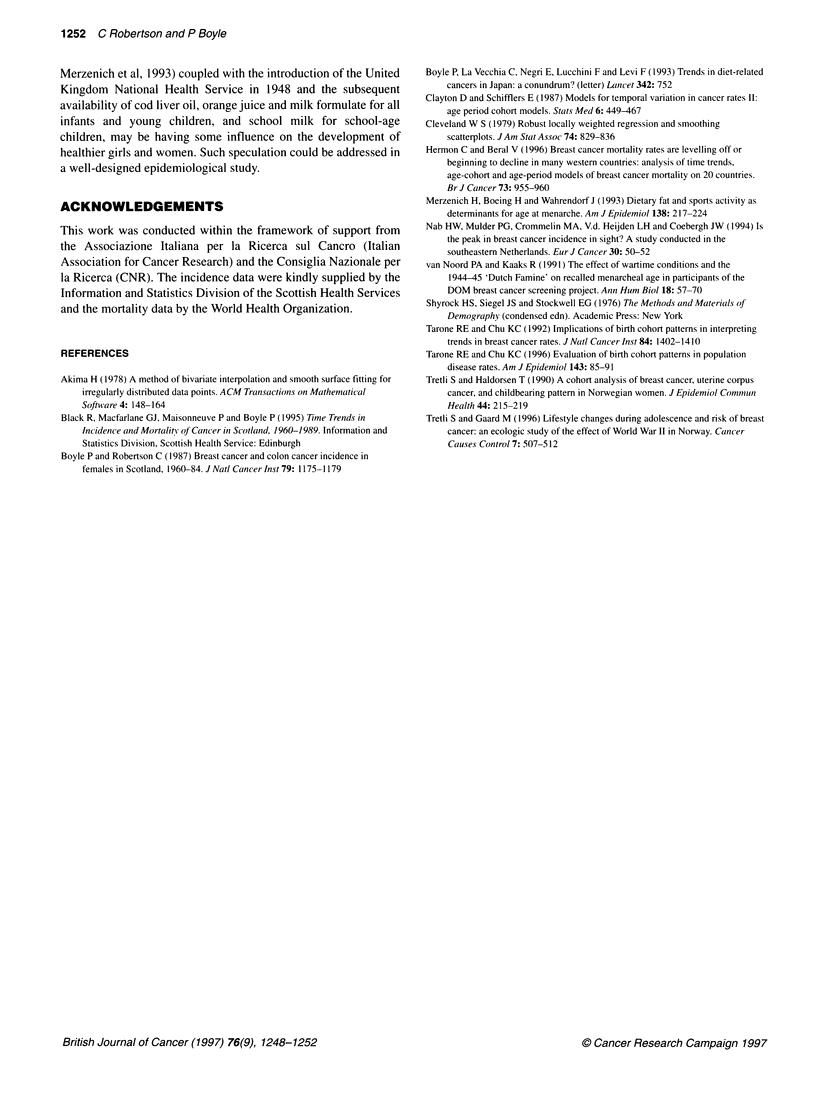# Statistical modelling of breast cancer incidence and mortality rates in Scotland.

**DOI:** 10.1038/bjc.1997.542

**Published:** 1997

**Authors:** C. Robertson, P. Boyle

**Affiliations:** Division of Epidemiology and Biostatistics, European Institute of Oncology, Milan, Italy.

## Abstract

The interpretation of time trends in disease rates can be facilitated using estimable contrasts from age-period-cohort models. Cohort and period trends in breast cancer incidence and mortality rates in Scotland were investigated using contrasts that measure the changes in the linear trends. These contrasts were compared with estimates obtained from mortality rates in the USA and Japan. A significant moderation of both breast cancer incidence and mortality rates was observed in Scotland, associated with cohorts of women born after the Second World War compared with women born between the two world wars. The moderation of breast cancer mortality among cohorts born after 1925 compared with cohorts born before 1925 that was observed in the USA and Japan was also observed in this study. This moderation is not present in the incidence rates. The relative decline in the risk of breast cancer seen in younger cohorts seems to be contradictory to the temporal pattern present among breast cancer risk factors. It may well be that the alteration of eating patterns as a result of rationing in the wartime and immediate post-war period, and the subsequent influence on certain breast cancer risk factors probably produced by such changes, may have had some influence on the development of healthier girls and women. Such speculation could be addressed in a well-designed epidemiological study. There have been no changes in the mortality rate trends with period in Scotland, although the changes in the incidence rate trends with period are consistent with an increase in registration coverage.


British Journal of Cancer (1997) 76(9), 1248–1252
© 1997 Cancer Research Campaign

### Statistical modelling of breast cancer incidence and mortality rates in Scotland

**C Robertson and P Boyle**

Division of Epidemiology and Biostatistics, European Institute of Oncology, Via Ripamonti 435, 20141 Milan, Italy

**Summary** The interpretation of time trends in disease rates can be facilitated using estimable contrasts from age–period–cohort models. Cohort and period trends in breast cancer incidence and mortality rates in Scotland were investigated using contrasts that measure the changes in the linear trends. These contrasts were compared with estimates obtained from mortality rates in the USA and Japan. A significant moderation of both breast cancer incidence and mortality rates was observed in Scotland, associated with cohorts of women born after the Second World War compared with women born between the two world wars. The moderation of breast cancer mortality among cohorts born after 1925 compared with cohorts born before 1925 that was observed in the USA and Japan was also observed in this study. This moderation is not present in the incidence rates. The relative decline in the risk of breast cancer seen in younger cohorts seems to be contradictory to the temporal pattern present among breast cancer risk factors. It may well be that the alteration of eating patterns as a result of rationing in the wartime and immediate post-war period, and the subsequent influence on certain breast cancer risk factors probably produced by such changes, may have had some influence on the development of healthier girls and women. Such speculation could be addressed in a well-designed epidemiological study. There have been no changes in the mortality rate trends with period in Scotland, although the changes in the incidence rate trends with period are consistent with an increase in registration coverage.

**Keywords:** age–period cohort models; breast cancer; incidence; mortality

It is important to understand the mechanisms underlying changing patterns of cancer rates, and this is especially appropriate when trying to understand the temporal evolution of breast cancer incidence and mortality rates. Such rates are subject to such a range of influences, such as the effects of the introduction of large-scale mammographic screening programmes and treatment advances; such influences must be taken into consideration so that a clearer understanding of underlying trends may be obtained from mathematical modelling in which incidence and mortality are considered simultaneously.

Herman and Beral (1996) have investigated time trends in breast cancer mortality in 20 countries and they concluded that there was evidence of a decline in mortality in most countries, which can be attributed in part to period and in part to cohort effects. This analysis used age–period and age–cohort models but did not use a full age–period–cohort model. Consequently, they were not able to estimate the effects of period adjusting for cohort and vice versa.

Tarone and Chu (1996) recently published an analysis of birth cohort patterns in breast cancer mortality rates in the USA and Japan. They also developed a methodology for testing changes in the trends associated with birth cohort using identifiable contrasts of cohort effects by extending the estimable curvatures of Clayton and Schifflers (1987). In line with their previous work (Tarone and Chu, 1992), they also advocated the use of 2-year age groups and time periods to reduce the overlap in the birth cohorts.

*Received 25 February 1997*
*Revised 27 May 1997*
*Accepted 30 May 1997*

*Correspondence to:* C Robertson

The main conclusion of Tarone and Chu (1996) was that there was evidence in both the USA and the Japanese mortality data of a change in the cohort trend around 1925. Tarone and Chu (1996) suggested that it would be useful to determine the extent to which this moderation in breast cancer mortality risk with birth cohorts beginning in the mid 1920s is a common feature in international data. In addition, they argued that there was little scope for any different conclusions based on an analysis of incidence rates as improvements in breast cancer survival were unlikely to have had a major impact on mortality rates.

Here, we apply the analysis philosophy of Tarone and Chu (1996) to the situation in Scotland where long time series of cancer mortality and cancer incidence data are both available for the entire national population (Black et al, 1995). It is the purpose of this study to (1) extend the international comparison of breast cancer mortality rates using the methodology of Tarone and Chu (1996); (2) to extend these comparisons to incidence and mortality rates from the same country; and (3) investigate if changes in mortality rates are consistent with those for incidence rates. This can only be accomplished in countries, such as Scotland, for which both incidence and mortality data are routinely collected.

### MATERIALS AND METHODS

Cancer mortality and population data for Scotland are available for 5-year age groups from 1950 to 1990 from the World Health Organization mortality database, and individual records of cancer incidence are available from 1960 to 1990 (Black et al, 1995). These data give an opportunity to compare simultaneously the changes in cohort patterns in both incidence and mortality.

Using the individual records of all incident cases of breast cancer diagnosed in Scotland between 1960 and 1989, a two-way

**Figure 1** Estimated birth cohort effects for breast cancer incidence rates in women in Scotland 1960–89. The estimates on the *y*-axis are log-relative risks table of age group by time period was constructed using intervals of 2 years. All ages from 20 to 83 years were used giving 32 age groups, 15 time periods and 46 birth cohorts. The first cohort corresponds to those who were aged 82–83 years in 1960–62, i.e. born in 1877–80; the second cohort were aged 80–81 years in 1960–62, i.e. born in 1879–82. Thus there is some overlap of cohorts, and the convention used here is to take the central two years 1878–79, 1880–01, etc. and to refer to the cohort by the first of these 2 years.

The population data are only available for 5-year age groups for each year from 1950. For the analysis using 2-year age groups and time periods, it was necessary to interpolate the populations in single years of age, and this was achieved by using a smoothed two-dimensional cubic interpolation (Akima, 1978). This is not as sophisticated as Beer's method (Shyrock et al, 1976), used by Tarone and Chu (1996), as it does not guarantee that the 5-year totals are preserved. As a check on the interpolation, the 5-year totals based on the interpolation were compared with the data. The average absolute percentage difference was 0.35% and the maximum discrepancy was 1.37% occurring in the oldest age group. These differences are small and unlikely to lead to any great bias, as the number of incidence cases is small relative to the population sizes, which range from 208 000 among 20–24 year olds to 70 000 in the 80–84 age group in 1989.

For the analysis of mortality data, the standard 5-year age groups and time periods were used. In this instance, there are 13 age groups from 20–24 to 80–84 years, eight time periods from 1950–89 and hence 20 cohorts, denoted by their mid years: 1870, 1875, etc.

The parameters of the age–period–cohort model were estimated using a generalized linear model with binomial errors and a logistic link. With the incidence data, no cases were observed in cohorts 45 (1966–67) and 46 (1968–69). The three points corresponding to these cohorts were not used in the analysis as the estimates of the corresponding parameters would tend to negative infinity. A constraint was necessary to obtain parameter estimates because of the linear dependency between age, period and cohort, and here the first and last cohort were constrained. As this analysis uses identifiable contrasts, the choice of constraint in the full age–period–cohort model was not important as the same estimated value of the contrast was obtained whichever constraint was used.

In the usual age–period–cohort model for $n_a$ age groups and $n_p$ time periods, the expected value of the disease rate ($E_{ij}$) is expressed as a linear combination of the effects of age, period and cohort: $\ln(E_{ij}) = \mu + \alpha_i + \pi_j + \gamma_k$, where $\mu$ is the intercept, $\alpha_i$ is the effect of age group $i$, $\pi_j$ is the effect of time period $j$ and $\gamma_k$ is the effect of birth cohort $k = j - i + n_a$. The estimates of $\alpha_i$, $\beta_j$ and $\gamma_k$ were not identifiable as different estimates were obtained depending on the identifiability constraint used; however, Tarone and Chu (1996) showed that linear contrasts that estimated the difference in trends in two distinct eras were identifiable. The analysis presented here is based on the use of such contrasts.

One of the main aims of the analysis is to compare the trend in the eight cohorts born just after the turn of the century to the eight cohorts born from 1924 onwards, and an appropriate contrast comparing the linear trends in the two eras is:

$$\begin{aligned} C^8_{1900,1924} = {} & 7\gamma_{1938} + 5\gamma_{1936} + 3\gamma_{1934} + 1\gamma_{1932} - 1\gamma_{1930} - 3\gamma_{1928} \\ & - 5\gamma_{1926} - 7\gamma_{1924} - (7\gamma_{1914} + 5\gamma_{1912} + 3\gamma_{1910} + 1\gamma_{1908} \\ & - 1\gamma_{1906}\ 3\gamma_{1904} - 5\gamma_{1902} - 7\gamma_{1900}) \end{aligned}$$

This is the same as contrast $C_2$ of Tarone and Chu (1996). If the estimated value of this contrast is zero, then the linear trend in the cohort effects among the 1900–14 cohort era is the same as that in the 1924–38 era. If $C^8_{1900,1924}$ is negative, then the trend in the younger cohort era is not as steep as in the older cohort era, and this would provide evidence that there had been a moderation of breast cancer risk among the younger cohort era. This is not interpreted as a reduction in risk, although this may have happened, but merely that the rate of increase in risk is not as steep.

In the notation adopted here for the contrast $C^m_{y,z}$, $y$ is the beginning of the first cohort era, $z$ is the beginning of the second cohort era and $m$ is the number of cohorts in each era. These cohorts are all of equal width ($w$ years) and so the first era is from $y$ to $y + w(m - 1)$ with the second from $z$ to $z + w(m - 1)$. This uses the convention that the cohort is referred to by the first of the two central years in it. The difference between $y$ and $z$ must be at least $w(m + 1)$ years to avoid any overlap in the cohort eras. Similar contrasts can be defined for periods, and these are denoted $P^m_{y,z}$, where $y$ is the beginning of the first period block, $z$ is the beginning of the second period block and $m$ is the number of periods in each block. If the periods are all of width $w$ years, then the difference between $y$ and $z$ must be at least $wm$ years to avoid any overlap in the period blocks. This is different from the cohort eras, as the periods have no overlap and there is overlap in the cohorts.

### RESULTS

The age–period–cohort model of the incidence rates had a deviance of 513 on 389 degrees of freedom. Residual analysis revealed that the lack of fit was associated with overdispersion. The model for the mortality rates had a deviance of 65 on 66 degrees of freedom. For incidence, there was evidence of significant non-linear period and cohort effects, allowing for overdispersion, but for mortality only the non-linear cohort effects were significant. The estimated cohort effects are plotted for incidence and for mortality in Figure 1 and Figure 2, respectively, together with a locally weighted smooth line (Cleveland, 1979). These estimates were obtained under the assumption that the first and last cohorts had the same effect. Consequently the effect for the last cohort is plotted at zero, and the points plotted are subject to this arbitrary restriction. Changes in incidence in the cohort trends are

© Cancer Research Campaign 1997

**Figure 2** Estimated birth cohort effects for breast cancer mortality rates in women in Scotland 1950–89. The estimates on the *y*-axis are log-relative risks clearly visible at around 1936. This is about 10 years later than the pattern in the USA mortality rates. There are two changes of slope in the cohort effects for the Scottish mortality data. One takes place about 1890 and the other about 1925. The Scottish mortality data and incidence data both cover a longer period than the USA mortality data, and there is no information from the USA on cohorts born before 1887.

Tarone and Chu (1996) investigated eight cohorts in two eras: cohorts born in 1900–14 and cohorts born in 1924–38. Using the USA data, the estimated contrast for comparing the slopes was $C^8_{1900,1924} = -4.157$ (s.e. = 0.175). This contrast compares the slope in the latter era with that in the former. As the Japanese data were only available as 5-year age groups and time periods, there are only four cohorts in each of the two eras, and the appropriate contrast for comparing the two eras is $C^4_{1900,1925} = -0.568$ (s.e. = 0.123). Both of these contrasts suggest that there was a moderation of breast cancer mortality risk trend in both the USA and Japan beginning with women born after 1925 relative to those born in 1900–25.

If the two eras 1900–14 and 1924–38 are considered and the same cohort contrast is estimated for the Scottish incidence data as for the USA mortality data in Tarone and Chu (1996), then the estimated value is $C^8_{1900,1924} = -0.52$ (s.e. = 0.42), which does not provide any evidence for changes in cohort trend. The major event of the last 50 years was the Second World War, and it is of interest to investigate whether there was any change in the cohort trends before and after the war. The trend in the era 1946–60 was compared with that in 1924–38. The estimated value is $C^8_{1924,1946} = -4.76$ (s.e. = 1.77), which is consistent with a major change in cohort-based trends in breast cancer incidence.

**Table 1** Cohort contrasts for incidence and mortality

| Incidence | | | Mortality | | |
|---|---|---|---|---|---|
| **Initial years of the two eras** | **Estimate** | **s.e.** | **Initial years of the two eras** | **Estimate** | **s.e.** |
| | | | 1875,1900 | 0.76 | 0.15 |
| 1880,1904 | –0.63 | 0.86 | 1880,1905 | 0.48 | 0.12 |
| 1882,1906 | –1.49 | 0.72 | | | |
| 1884,1908 | –0.48 | 0.61 | | | |
| 1886,1910 | 0.11 | 0.54 | 1885,1910 | 0.38 | 0.11 |
| 1888,1912 | 0.10 | 0.49 | | | |
| 1890,1914 | 0.19 | 0.46 | 1890,1915 | 0.28 | 0.11 |
| 1892,1916 | 1.15 | 0.44 | | | |
| 1894,1918 | 1.82 | 0.42 | | | |
| 1896,1920 | 0.09 | 0.41 | 1895,1920 | –0.01 | 0.12 |
| 1898,1922 | –0.14 | 0.41 | | | |
| 1900,1924 | –0.52 | 0.41 | 1900,1925 | –0.43 | 0.14 |
| 1902,1926 | –0.77 | 0.43 | | | |
| 1904,1928 | 0.28 | 0.45 | | | |
| 1906,1930 | –0.94 | 0.47 | 1905,1930 | –0.62 | 0.18 |
| 1908,1932 | –1.69 | 0.51 | | | |
| 1910,1934 | –1.09 | 0.54 | 1910,1935 | –0.72 | 0.26 |
| 1912,1936 | –2.76 | 0.63 | | | |
| 1914,1938 | –2.74 | 0.73 | | | |
| 1916,1940 | –4.40 | 0.88 | 1915,1940 | –1.03 | 0.44 |
| 1918,1942 | –4.81 | 1.07 | | | |
| 1920,1944 | –3.48 | 1.32 | 1920,1945 | –2.91 | 1.13 |
| 1922,1946 | –4.62 | 1.77 | | | |
| 1924,1948 | –2.81 | 2.12 | | | |

The magnitudes of the estimates are not directly comparable as the contrasts for the incidences are based on cohort eras with eight cohorts each of 2 years whereas for the mortality data the cohort eras have four cohorts each of 5 years. The first contrast for the incidence rates is $C^8_{1880,1904}$, which compares the trend in the 1880–94 era with that in the 1904–18 era. For the mortality rates, the first contrast is $C^4_{1875,1900}$, which is a comparison of the trend in the 1875–95 era with that in the 1900–20 era.

 © Cancer Research Campaign 1997

Different contrasts had to be used for the mortality data, as the data were only available in 5-year periods, although the same two eras were compared. Tarone and Chu (1996) compared the four cohorts centred on 1900, 1905, 1910 and 1915 with the four centred on 1925, 1930, 1935 and 1940 using the contrast that they denoted as $C_3$. The estimated value for the Scottish mortality data is $C^4_{1900,1925} = -0.43$ (s.e. = 0.15), which is similar to that reported by Tarone and Chu (1996) for the Japanese mortality data. For the pre- and post-war comparison, the estimated value is $C^4_{1920,1945} = -2.91$ (s.e. = 1.12). The four pre-war cohorts were centred on 1920–35, and the four post-war cohorts were centred on 1945–1960.

Similar results for both the post- and pre-war comparison and the post- and pre-1925 comparison of mortality rates were obtained when using eras of only three cohorts. In this instance, the contrasts of the three cohorts have coefficients of (–1, 0, 1), similar to the period contrast in Tarone and Chu (1996). The estimate of the comparison of the 1900–10 cohorts with the 1925–35 cohorts is $C^3_{1900,1925} = -0.085$ (s.e. = 0.039), and that for comparing 1925–35 with 1950–60 is $C^3_{1925,1950} = -0.84$ (s.e. = 0.37). Thus, the conclusions about the existence of a change in trend in mortality rates were not seriously influenced by the choice of cohort eras; however, the estimated magnitude of the change was influenced.

Unlike the situation reported in the USA, with respect to mortality in Scotland, there was no evidence of any increase in the period slopes in the 1980s compared with the 1970s $P^2_{1972,1982} = 0.01$ (s.e. = 0.03). There was a reduction in the linear trend in the incidence rates with period for the incidence data after 1975 compared with before 1975, $P^6_{1962,1978} = -0.97$ (s.e. = 0.19). This has been explained previously as being most probably due to an increase in registration coverage (Boyle and Robertson, 1987).

In this study, the cohort contrasts were specified and linked to stated hypotheses; this reduces the problem of multiple testing. It is possible to adopt a sliding scale for the cohort contrasts and move the contrast across the cohorts to investigate the consistency of the contrasts over time. Each cohort contrast is indexed by the initial year of the two cohort eras and the number of cohorts in each era. Thus, there are many possible sliding contrasts to use. It is not sensible to have too large a gap between the two eras when looking for local changes in trend. The incidence contrasts, $C^8_{y,(y+24)}$, where $y$ = 1880,1882 ... 1924, and the sliding contrasts for the mortality rates, $C^4_{y,(y+25)}$ where $y$ = 1875,1880 ... 1920, are presented in Table 1.

Statistical inferences for these contrasts are difficult as they are not independent of each other, and many quantities estimating similar changes in trend are presented. However, these contrasts have a descriptive value, and it can be seen that the pattern of the changes in the cohort trends for incidence are similar to those for mortality except for cohorts born before 1900. The earlier cohorts are the ones who contribute most to the mortality rates in the early periods for which under-registration may be a problem.

### DISCUSSION

The analysis presented here benefits from modelling incidence and mortality data and testing the same effects for both rates. The replication of previously published identifiable contrasts in different countries provides valuable evidence on the stability of the effects. As this analysis uses only identifiable contrasts, it does not suffer from the assumptions about the lack of period or cohort effects that are implicit in the analysis of Hermon and Beral (1996). This method provides estimates that are readily interpretable and permit valid comparisons.

When our analysis is compared with the recent analysis of data from the USA and Japan by Tarone and Chu (1996), it is clear that there are similar changes in the cohort trends in the mortality rates around 1925 in Scotland, the USA and Japan. There is a decrease in the slope observed in all three countries. However, it is interesting that this same pattern of reduction is not observed in the Scottish incidence data. Rather than showing a clear difference in the eras before and after 1925, examination of the Scottish incidence and mortality data shows evidence of a decrease in the cohort trends associated with those born after the Second World War compared with those born before. This trend can also be seen in the younger cohorts of the USA mortality data (Tarone and Chu, 1996; Figure 1), but it is not present in the Japanese data in which a small increase is suggested; this could be due in some small part to the westernization of the Japanese diet subsequent to 1945 (Boyle et al, 1993). The increase in birth cohort mortality around the turn of the century, estimated here in Table 1 and visible in Figure 2, is present in all three countries.

In Scotland, cohort-based changes in breast cancer mortality rates began about 20 years before any cohort-based changes in incidence rates. The changes in the period and age trends were similar for both incidence rates and mortality rates in Scotland. This may be explained by poor registration in the early periods, particularly among the older age groups. It could also be explained by gradual treatment improvements from the late 1970s onwards among post-menopausal breast cancers: this would coincide with growth in the use of chemotherapy and, particularly, tamoxifen, which have been shown to increase breast cancer survival when used as an adjuvant therapy. Tamoxifen is more effective among post-menopausal women with the disease (Early Breast Trialists Group, 1994).

The analysis presented here benefits from being based on both cancer mortality and cancer incidence data that are collected on the national population of Scotland by two independent data collection schemes: mortality data from the Registrar General for Scotland, who is responsible for all vital statistics schemes, and cancer incidence data from the National Cancer Registration scheme. Examination of breast cancer incidence data during this time period is free from the major influence of the implementation of the national breast cancer screening programme available to all women in Scotland every three years from 1989 onwards. After that point, an increase in incidence would be expected at ages 50 to 69 years because of the impact of screening, and a decrease in mortality rates in the same age groups is to be hoped for.

The relative decline in the risk of breast cancer seen in younger cohorts of women in Scotland has been reported before (Boyle and Robertson, 1987) and seems to be contradictory to the temporal pattern present for breast cancer risk factors: women having a continually earlier age at menarche, fewer women having children, the average number of births decreasing, the age at first birth increasing and a diet higher in salt and fat in adult life and around the menarcheal period. Changes in the Second World War Cohort have also been observed in Norway (Tretli and Haldorsen, 1990; Tretli and Gaard, 1996), mainly for endometrial cancer risk factors, and in the Netherlands (van Noord and Kaaks, 1991; Nab et al, 1994). It may well be that the alteration of eating patterns as a result of rationing in the wartime and immediate post-war period, and the subsequent influence on certain breast cancer risk factors probably produced by such changes (e.g. age at menarche;

© Cancer Research Campaign 1997 

Merzenich et al, 1993) coupled with the introduction of the United Kingdom National Health Service in 1948 and the subsequent availability of cod liver oil, orange juice and milk formulate for all infants and young children, and school milk for school-age children, may be having some influence on the development of healthier girls and women. Such speculation could be addressed in a well-designed epidemiological study.

### ACKNOWLEDGEMENTS

This work was conducted within the framework of support from the Associazione Italiana per la Ricerca sul Cancro (Italian Association for Cancer Research) and the Consiglia Nazionale per la Ricerca (CNR). The incidence data were kindly supplied by the Information and Statistics Division of the Scottish Health Services and the mortality data by the World Health Organization.

### REFERENCES

Akima H (1978) A method of bivariate interpolation and smooth surface fitting for irregularly distributed data points. *ACM Transactions on Mathematical Software* **4:** 148–164

Black R, Macfarlane GJ, Maisonneuve P and Boyle P (1995) *Time Trends in Incidence and Mortality of Cancer in Scotland, 1960–1989*. Information and Statistics Division, Scottish Health Service: Edinburgh

Boyle P and Robertson C (1987) Breast cancer and colon cancer incidence in females in Scotland, 1960–84. *J Natl Cancer Inst* **79:** 1175–1179

Boyle P, La Vecchia C, Negri E, Lucchini F and Levi F (1993) Trends in diet-related cancers in Japan: a conundrum? (letter) *Lancet* **342:** 752

Clayton D and Schifflers E (1987) Models for temporal variation in cancer rates II: age period cohort models. *Stats Med* **6:** 449–467

Cleveland W S (1979) Robust locally weighted regression and smoothing scatterplots. *J Am Stat Assoc* **74:** 829–836

Hermon C and Beral V (1996) Breast cancer mortality rates are levelling off or beginning to decline in many western countries: analysis of time trends, age-cohort and age-period models of breast cancer mortality on 20 countries. *Br J Cancer* **73:** 955–960

Merzenich H, Boeing H and Wahrendorf J (1993) Dietary fat and sports activity as determinants for age at menarche. *Am J Epidemiol* **138:** 217–224

Nab HW, Mulder PG, Crommelin MA, V.d. Heijden LH and Coebergh JW (1994) Is the peak in breast cancer incidence in sight? A study conducted in the southeastern Netherlands. *Eur J Cancer* **30:** 50–52

van Noord PA and Kaaks R (1991) The effect of wartime conditions and the 1944–45 'Dutch Famine' on recalled menarcheal age in participants of the DOM breast cancer screening project. *Ann Hum Biol* **18:** 57–70

Shyrock HS, Siegel JS and Stockwell EG (1976) *The Methods and Materials of Demography* (condensed edn). Academic Press: New York

Tarone RE and Chu KC (1992) Implications of birth cohort patterns in interpreting trends in breast cancer rates. *J Natl Cancer Inst* **84:** 1402–1410

Tarone RE and Chu KC (1996) Evaluation of birth cohort patterns in population disease rates. *Am J Epidemiol* **143:** 85–91

Tretli S and Haldorsen T (1990) A cohort analysis of breast cancer, uterine corpus cancer, and childbearing pattern in Norwegian women. *J Epidemiol Commun Health* **44:** 215–219

Tretli S and Gaard M (1996) Lifestyle changes during adolescence and risk of breast cancer: an ecologic study of the effect of World War II in Norway. *Cancer Causes Control* **7:** 507–512

© Cancer Research Campaign 1997